# Dietary restriction rescues 5-fluorouracil-induced lethal intestinal toxicity in old mice by blocking translocation of opportunistic pathogens

**DOI:** 10.1080/19490976.2024.2355693

**Published:** 2024-05-23

**Authors:** Duozhuang Tang, Rongrong Qiu, Xingxing Qiu, Man Sun, Mingyue Su, Zhendong Tao, Liu Zhang, Si Tao

**Affiliations:** a Jiangxi Key Laboratory of Clinical and Translational Cancer Research, Department of Oncology, The Second Affiliated Hospital of Nanchang University, Nanchang, Jiangxi, China; b Department of Hematology, The Second Affiliated Hospital of Nanchang University, Nanchang, Jiangxi, China; c Department of Medical Laboratory Medicine, Jiangxi Province Hospital of Integrated Chinese & Western Medicine, Nanchang, Jiangxi, China; d Intensive Care Unit, Beijing Jishuitan Hospital, Capital Medical University, Beijing, China

**Keywords:** Dietary restriction, chemotherapy, aging, intestinal toxicity, gut microbiota

## Abstract

Chemotherapy remains a major treatment for malignant tumors, yet the application of standard dose intensity chemotherapy is limited due to the side effects of cytotoxic drugs, especially in old populations. The underlying mechanisms of cytotoxicity and strategies to increase the safety and tolerance of chemotherapy remain to be explored. Using 5-fluorouracil (5-FU), a cornerstone chemotherapeutic drug, we demonstrate that the main cause of death in *ad libitum* (AL) fed mice after 5-FU chemotherapy was infection caused by translocation of intestinal opportunistic pathogens. We show that these opportunistic pathogens greatly increase in the intestine after chemotherapy, which was closely related to loss of intestinal lysozyme. Of note, two weeks of dietary restriction (DR) prior to chemotherapy significantly protected the loss of lysozyme and increased the content of the beneficial *Lactobacillus* genera, resulting in a substantial inhibition of intestinal opportunistic pathogens and their translocation. The rescue effect of DR could be mimicked by Lysozyme or *Lactobacillus* gavage. Our study provides the first evidence that DR achieved a comprehensive protection of the intestinal physical, biological and chemical barriers, which significantly improved the overall survival of 5-FU-treated mice. Importantly, the above findings were more prominent in old mice. Furthermore, we show that patients over 65 years old have enriched opportunistic pathogens in their gut microbiota, especially after 5-FU based chemotherapy. Our study reveals important mechanisms for the poor chemotherapy tolerance of the elderly population, which can be significantly improved by short-term DR. This study generates new insights into methods for improving the chemotherapeutic prognosis by increasing the chemotherapy tolerance and safety of patients with malignant tumors.

## Introduction

Despite the emergence of many new anti-tumor drugs, at present, chemotherapy is still the foundation of malignant tumor treatment. However, the side effects induced by the cytotoxicity of chemotherapeutic drugs make it difficult for many patients to tolerate and persist in completing their treatment regimen, which is especially common in elderly patients.^1^ Currently, the aging of many populations is a serious challenge faced by many countries in the world. The incidence of malignant tumors increases significantly with age. Studies have anticipated that in 2030, more than 60% of patients diagnosed with most types of malignant tumors in the United States will be older than 65 years.^2–4^ Notably, compared with young patients, elderly patients who receive standard dose intensity chemotherapy find treatment more difficult to tolerate and are more prone to serious complications.^5–7^ This not only leads to a significant increase in hospitalization costs for elderly patients with malignant tumors, but also leads to poor overall prognosis. Therefore, it is of great importance to explore ways to improve the chemotherapy tolerance of patients with malignant tumors, especially for the elderly populations.

Under homeostatic conditions, the intestine of old mice exhibits mild histological changes such as a moderate increase in crypt length, Paneth cells, goblet cells, and a decrease in crypt number. Despite the increase in Paneth cell number with age, the transcript levels of lysozyme (an exclusive product of Paneth cells) and those of the Defa20 (a member of the defensins group produced exclusively by Paneth cells) are significantly reduced in the intestine of old mice.^5,8^ It has also been shown that the composition of gut microbiota is altered in old mice, which contributes to the increased systemic inflammation observed during aging.^9^ Intestinal permeability has been found to increase with age in both Drosophila and mice.^9,10^ In an in vitro culture system, we and others have reported that intestinal crypts derived from old mice exhibited impaired organoid formation.^11–13^ In an irradiation model, old mice exhibited impaired regeneration of their intestinal epithelial cells.^14^ Chemotherapeutic drugs frequently damage rapidly proliferating intestinal epithelial cells and induce intestinal mucositis and dysbiosis, both of which have been identified as important systems affecting multiple physiological and pathological processes.^15^

Gut microbiota plays a role in the onset and development of malignancies.^16^ Gut microbiota are also intimately linked to the pharmacological effects of chemotherapies including 5-FU and could modulate their therapeutic efficacy and toxicity.^16,17^ Probiotics, prebiotics, and synbiotics can prevent mucositis and reduce the occurrence of sepsis during chemotherapy. However, studies have shown that different chemotherapy regimens may lead to distinct alterations in the gut microbiota composition.^15,18^ It is unclear which specific bacteria in the gut microbiota of patients receiving 5-FU chemotherapy may play a key role in the treatment related toxicity, and these specific microbiota need to be explored.^16,17^ In particular, the effects of aging on chemotherapy-indued intestinal damage, regeneration, and gut microbiota dysbiosis, as well as their interaction and overall impact on the prognosis have not been well characterized.

Dietary restriction (DR) is a proven regimen to ameliorate aging-associated intestinal pathology across many species.^19–22^ Previously, we have shown that two weeks of DR before chemotherapy can significantly increase Lactobacillales in the intestinal microbiota and alleviate lethal-dose methotrexate (MTX)-induced intestinal inflammation. As a result, DR reduced intestinal damage and increased the survival rate of MTX-treated young mice.^23^ However, how DR achieved the modulation of gut microbiota and how intestinal barriers were regulated by DR in the context of chemotherapy was largely unknown. In particular, whether DR could increase the safety and improve the tolerance of chemotherapy in the elderly population and the underlying mechanism has not been studied.

MTX is less commonly used in the treatment of solid tumors in clinical practice with an indication only in breast cancer, gestational choriocarcinoma, and malignant hydatidiform mole, the later two of which have very low incidences. 5-FU is a pivotal chemotherapeutic drug in the treatment of malignant tumors, which is widely used in colorectal cancer, gastric cancer, breast cancer, lung cancer and other common malignant tumors. 5-FU kills rapidly proliferating cells by interfering with DNA replication by inhibiting nucleotide synthesis.^24,25^ As a result, tumor cells as well as healthy cells that proliferate rapidly – such as intestinal epithelial and hematopoietic cells – are damaged by 5-FU. These cytotoxicities could induce intestinal damage and myelosuppression, which is the major factor that limits patient tolerance to 5-FU.^26,27^

In this study, a model of 5-FU chemotherapy was established in young and old C57BL/6 mice, and the effect of short-term 30% DR before chemotherapy on survival and intestinal pathology was investigated. In particular, we performed a detailed study on old mice. The study shows that DR can significantly protect against the loss of lysozyme and increase the content of *Lactobacillus*, resulting in a significant inhibition of intestinal opportunistic pathogens and their translocation. DR achieved a comprehensive protection of the intestinal mechanical, biological and chemical barriers, which significantly improved the overall survival of mice. The study presents DR as a novel way to potentially improve the patient prognosis, by improving the chemotherapy tolerance and safety of patients with malignant tumors.

## Materials and methods

### Mice

Female C57BL/6j mice were purchased from Hunan SJA Laboratory Animal Co., Ltd. and maintained in the animal facilities of Nanchang Royo Biotech under pathogen-free conditions on a 12-h light/12-h dark cycle. 2-month-old mice were used as the young group, and 20- to 24-month-old mice were used as the old group. DR were performed as previously described.^28,29^ Age and bodyweight matched mice were randomly divided into either the AL-fed or DR-fed group by the researchers. Randomization according to the ARRIVE Guidelines was not performed when establishing experimental groups. Mice were housed individually so that the daily food consumption of each mouse could be determined. This was measured every day for one week to determine their AL-feeding rate. After the 1-week measurement, the average amount of food was determined for every mouse. During the feeding protocol, the AL mice were fed with unlimited access of food, while DR mice were fed with 70% the average amount of food according to the previous calculation. The calculated 70% food pellet was added to each cage daily at the same time, and was constant over the whole DR period. For the young mouse group, 2 months old mice were used; for the old mouse group, 20–24 months old mice were used. All mouse experiments were approved by the Animal Experimental Ethical Inspection of Nanchang Royo Biotech Co. Ltd (RYE2022041301).

### Diarrhea assessment

Bowen scoring system was used to determine a diarrhea score for each mouse. The severity of diarrhea was scored using the following scale, 0: normal (normal stool); 1: minimal (soft stool); 2: slight (slightly wet and soft stool); 3: moderate (wet and unformed stool with moderate perianal a staining of the coat); 4: severe (watery stool with severe perianal staining of the coat). The incidence of each diarrhea score (0–4) and the diarrhea score were used to evaluate the severity of diarrhea.^30,31^ Diarrhea assessment was performed by one blind investigator.

### Criteria for defining death or moribundity

Criteria for defining Death or Moribundity according to the Guidelines for Endpoints in Animal Study Proposals include: lack of responsiveness to manual stimulation; immobility; and/or an inability to eat or drink; abnormal posture, rough hair coat, exudates around eyes, abnormal breathing, difficulty with ambulation. Mice were recorded as non-survived when they meet the above criteria. Mice which did not meet the criteria were recorded as survived.

### Histology

Intestinal cross-sections through intestinal rolls were prepared as previously described.^32^ 3-micrometer paraffin sections of the intestine were used for H&E staining. For immunohistochemistry staining, 5-micrometer paraffin sections of intestine samples were used. The sections were deparaffinized and hydrated, and then placed in an antigen retrieval solution (0.01 M citrate buffer, pH 6.0) for 15 min in a microwave oven at 100°C at 600 W. The antigen retrieval solution is placed at room temperature for about 30 min, cooled to room temperature, and the sections are taken out and rinsed and incubated with freshly prepared 3% H_2_O_2_ for inhibition of endogenous peroxidase. Then the sections were washed and afterward incubated with primary antibody at 4°C overnight in a humid chamber. The slides were then washed three times with PBS the next day, and incubated with goat anti-mouse biotinylated IgG at room temperature for 1 hour. Afterward, the slides were washed and stained with DAB and were counterstained with hematoxylin, dehydrated, and mounted. For a negative control in the IHC procedures performed, mouse 10% normal serum replaced the primary antibodies. Antibodies used were the following: Olfm4 (Cell Signaling Technology, Cat.#39141) 1:300 diluted; lysozyme (Sigma, Cat.L-6876) 1:4000 diluted; Goat anti-mouse biotinylated IgG (ZSGB-BIO, PV-6000) 1:1000 diluted.

5-micrometer paraffin sections of the intestine were used for TUNEL staining (KeyGEN BioTECH, Cat.KGA7071). The sections were deparaffinized and hydrated, and then incubated at room temperature with 20 μg/ml proteinase K for 20 min. The sections were washed and subsequently incubated with 3% hydrogen peroxide in absolute methanol for 5 min at room temperature to inhibit endogenous peroxidase. Then the sections were washed and afterward incubated in blocking buffer (10% BSA in PBS) for 10 min. After being rinsed in PBS, the sections were incubated in TdT equilibration buffer for 5 min. Slides were incubated in the TUNEL mixture for 60 min at 37°C afterward and washed in PBS. Then slides were mounted with mounting medium with DAPI (Vector) and covered with coverslips.

All the images were taken using the OLYMPUSIX73 microscope (Japan, Inverted Fluorescences microscope). For villi frequencies, five visual fields were randomly selected per sample and villi number in 1 mm length as a unit was counted. For villi height, 45 villi were counted in each sample. For the ratio of villi height versus crypt depth (VH/CD), 45 villi and neighboring crypts were counted in each sample. For the number of basal crypt Olfm4-positive cells and lysozyme-positive cells, 30 crypts were counted in each sample. Crypt apoptotic cell number in jejunum was determined by TUNEL staining with 30 crypts counted for each sample.

### Methodology for toxicity evaluation

Toxicity of 5-FU was evaluated referring to OECD (Organisation for Economic Co-operation and Development) reporting guidelines. Bodyweight of every mouse was measured and recorded daily before and until day 15 after 5-FU treatment. The bodyweight records for young and old, AL and DR, before and after 5-FU treatment are shown in Supplementary table S1. Diarrhea assessment was done every other day until day 8 after 5-FU treatment by using the Bowen scoring system as described above. Histopathological analyses were performed on intestinal sections at indicated time-points and the staining method was described in the Histology section.

### Drug administration

#### 5-FU treatment

5-FU was purchased from Tianjin Jinyao Pharmaceutical Co., Ltd, China (CAS n°: 51-21-8; Lot number: 2211141; purity: 99%), and was diluted in saline for injection. Mice were injected intraperitoneally with 5-FU for 5 days (day-4, day0). The daily dose of 5-FU was 50 mg/kg for young mice.^33,34^ To increase safety and tolerance, a 20% of dose reduction was applied in the old mice (40 mg/kg per day) as in clinical practices.^33^ In the control group, saline was injected instead of 5-FU.

#### Broad-spectrum antibiotic treatment

Broad-spectrum antibiotic treatment was performed according to previous publications.^35,36^ Mice were fed by way of gavage with 0.2 ml broad-spectrum antibiotics at a concentration of 10 mg/mice/day including ampicillin (Solarbio), neomycin (Solarbio), metronidazole (Solarbio) and vancomycin (Lilly, Inc.) for 5 days to ensure equal doses taken by each mouse, then the antibiotics administered in drinking water (ampicillin, neomycin, and metronidazole: 1 g/L; vancomycin: 500 mg/L) until the end of the experiment to avoid too much stress which would have been induced by long-term gavage. Drinking water was exchanged twice a week. For the control group, saline was administrated in the same way as the antibiotics.

#### Lactobacillus rhamnosus GG treatment

*L. rhamnosus GG* (LGG) (Beina Biotechnology, BNCC186192) were grown at 5% CO_2_ and 37°C in MRS (DeMan, Rogosa and Sharpe) medium (Solarbio, M8540). LGG was administered by gastric gavage at a dose of 1 × 10^9^ colony forming unit (CFU) per mouse per day for 3 weeks before 5-FU treatment.^37^ The control group received vehicle control (saline) alone.

#### Lysozyme treatment

Lysozyme (Sigma, L6876) was dissolved in saline and administered by gastric gavage at a dose of 150 mg/kg per mouse per day for 2 weeks before 5-FU treatment.^38^ Saline was applied instead of lysozyme in the control group.

### Bacteria identification from liver and spleen homogenates

Mice were sacrificed on day 4 after chemotherapy and the liver and spleen were collected immediately and homogenized in 2 ml saline. Then the liver and spleen homogenates were added to 10 ml Brain Heart Infusion Broth (BHI, Hopebio, HB8297–5) in aerobic or anaerobic conditions at 37°C, and cultured for 24 hours. Then, bacteria were taken by using an inoculating loop from the BHI culture medium and applied to BHI-agar plates using the streak method in aerobic or anaerobic conditions at 37°C, and cultured for 24 hours.^39^ The bacteria are fully dispersed on the surface of the agar plate, so that single bacteria colonies can grow out.

Bacterial colonies grown out from the liver and spleen homogenates were identified as previously published using a matrix-assisted laser desorption ionization time-of-flight mass spectrometry (MALDI-TOF MS) device.^40–42^ Briefly, single colonies were evenly spread on wells of the MALDI sample target and the sample was allowed to dry at room temperature for a few minutes. One microliter of matrix solution (2,5-dihydroxybenzoic acid, 80 mg/mL, 30% acetonitrile, 0.1% trifluoroacetic acid) was then added and allowed to co-crystallize with the sample. Afterwards, one microliter of α-Cyano-4-hydroxycinnamic acid was added and the sample was allowed to dry at room temperature for a few minutes. MALDI-TOF MS was performed with the MicroFlex LT mass spectrometer (Bruker Daltonics, Bremen, Germany), as instructed by the manufacturer. The spectra were analyzed by using the Flex Control 3.0 software and MALDI Biotyper DB Update_3.3.1.0 database library (Bruker Daltonics, Bremen, Germany). The identification of the tested strain corresponds to the species of the reference strain having the best match in the database. According to the manufacturer’s instructions, an identification result with a score < 1.7 is considered invalid. A score between 1.7 and 1.99 indicates good identification to the genus level and a score ≥ 2.0 indicates good identification to the species level. In the current study, we recorded the strain with a score ≥ 2.0.

### Fecal sample collection

Fresh fecal samples were directly collected from each mouse by positioning a microtube in the proximity of the anus of the mouse. Excreted fecal pellets were collected in microtubes on ice and stored at −80°C within 1 h until DNA isolation for 16S rRNA gene sequencing.

### Fecal DNA isolation

Fecal samples were weighed and total DNA was extracted with the DP328 Fecal Genome Extraction Kit (Tiangen Biotech) according to the manufacturer’s instructions. DNA concentration and purity were measured with Nanodrop 2000.

### RNA isolation and cDNA synthesis

Total RNA was isolated from freshly collected intestinal tissues by using RNA simple Total RNA Kit (TianGen Biotech,Cat.#DP419). Reverse transcriptions were performed to synthesize first strand DNA by using RevertAid First Strand cDNA Synthesis kit (Thermo scientific) according to the manufacturer’s instructions with a procedure of incubation at 42°C for 60 min and heating inactivation at 70°C for 5 min.

### Quantitative real-time PCR (qPCR)

For detection of gene expression in intestinal tissues, qRT-PCR was performed by using TransStart Tip Green qPCR SuperMix (TransGen Biotech) with an ABI 7900 Real-Time PCR system (Applied Biosystems) in triplicates. Relative expression of genes was normalized to β-actin in each sample and was normalized to 1 in the AL group. Primer sets are listed in Table S2.^28,43–45^

For quantitative determination of bacteria in fecal samples, qPCR assays were carried out by using Ace qPCR SYBR Green Master Mix kit (Vazyme) with Bio-RAD 9600A system following the manufacturer’s instructions. The copy number of target DNA was determined by serially diluting standards (10^4^ to 10^8^ copies of plasmid DNA containing the respective amplicon for each set of primers) running on the same plate. The bacterial quantity was expressed as copies per gram of stool. Primer sets are listed in Table S3.^46–48^

### 16S rRNA gene sequencing

Fecal-sample DNA was extracted using DNA extraction kit. The concentration and purity were measured using the NanoDrop One (Thermo Fisher Scientific, MA, USA). 16S rRNA gene regions V3-V4 were amplified using universal primers^49^ (mice: 338F 5ʹ-ACTCCTACGGGAGGCAGCA-3ʹ and 806 R 5ʹ-GGACTACHVGGGTWTCTAAT-3ʹ; human: 341F-806 R 341F 5ʹ-CCTAYGGGRBGCASCAG-3ʹ and 806 R 5ʹ-GGACTACNNGGGTWTCTAAT-3ʹ) with 12 bp barcode, Primers were synthesized by Invitrogen (Invitrogen, Carlsbad, CA, USA). PCR reactions, containing 25 μl 2 × Premix Taq (Takara Biotechnology, Dalian Co. Ltd., China), 1 μl each primer(10 μM) and 3 μl DNA (20 ng/μl) template in a volume of 50 µl, were amplified by thermocycling: 5 min at 94°C for initialization; 30 cycles of 30 s denaturation at 94°C, 30 s annealing at 52°C, and 30 s extension at 72°C; followed by 10 min final elongation at 72°C. The PCR instrument was BioRad S1000 (Bio-Rad Laboratory, CA, USA).

The length and concentration of the PCR products were detected by 1% agarose gel electrophoresis. PCR products were mixed in equimolar ratios according to the GeneTools Analysis Software (Version4.03.05.0, SynGene). Then, the PCR mixture was purified with EZNA Gel Extraction Kit (Omega, USA). Sequencing libraries were generated using NEBNext® Ultra™ DNA Library Prep Kit for Illumina® (New England Biolabs, USA) following the manufacturer’s recommendations and index codes were added. The library quality was assessed on the Qubit 2.0 Fluorometer (Thermo Scientific). Finally, the library was sequenced on an Illumina Nova6000 platform and 250 bp paired-end reads were generated.

### Sequencing data processing

#### Paired-end raw reads quality control

Fastp (version 0.14.1, https://github.com/OpenGene/fastp) was used to control the quality of the Raw Data by sliding window(-W 4 -M 20). The primers were removed by using cutadapt software (https://github.com/marcelm/cutadapt/) according to the primer information at the beginning and end of the sequence to obtain the paired-end clean reads.

#### Paired-end clean reads assembly

Paired-end clean reads were merged using usearch -fastq_mergepairs (V10, http://www.drive5.com/usearch/) according to the relationship of the overlap between the paired-end reads, when at least 16 bp overlap the read generated from the opposite end of the same DNA fragment, the maximum mismatch allowed in overlap region was 5 bp, and the spliced sequences were called Raw Tags.

#### Raw tags quality control

Fastp was used to remove low-quality sequences. The average quality values for the bases in the sliding window were calculated and the non-conforming sliding windows were removed. The -W parameter specifies the size of the sliding window, which is 4 by default, and the -M parameter is used to specify the required average quality value, which is 20 by default (Q20). Fastp (version 0.14.1, https://github.com/OpenGene/fastp) was used to control the quality of the raw Data by sliding window (-W 4 -M 20) to obtain the paired-end clean tags.

### OTU cluster and species annotation

OTU (operational taxonomic units) clustering was used to classify groups of closely related individuals. Sequences analysis was performed by usearch software (V10, http://www.drive5.com/usearch/). Sequences with ≥ 97% similarity were assigned to the same OTU. An OTU is thought to possibly represent a species. The most frequently occurring sequence was extracted as a representative sequence for each OTU and was screened for further annotation.

### Clinical specimen collection

Clinical specimens were collected from patients undergoing chemotherapeutic treatment at the Second Affiliated Hospital of Nanchang University (Age < 60y cohort, *n* = 11; Age > 65y cohort, *n* = 11). Use of human samples was approved by the Institutional Ethics Committee of the Second Affiliated Hospital of Nanchang University, Nanchang, China. All procedures performed in this study using human data were in accordance with the Declaration of Helsinki (as revised in 2013). Informed consent was waived by the Institutional Ethics Committee of the Second Affiliated Hospital of Nanchang University because of the retrospective nature of this study.

### Statistics

GraphPad Prism 9.0 software was used for statistical analysis. To calculate p-values, the unpaired two-tailed Student’s t test was used for two-group datasets and one-way ANOVA was used for multi-group (more than two groups) datasets. Gehan-Breslow-Wilcoxon test was used for survival rate analysis. All results were displayed as mean ±SD. ns, not significant; *p < .05; **p < .01; ***p < .001; ****p < .0001.

Power calculation was not performed. For the survival curves, sample sizes were determined referring to our previous publication.^23^ For other experiments with an exploratory objective, 5–6 mice per group per experiment were used.

## Results

### DR substantially increases survival rate of old mice treated with 5-FU

**Figure 1.**
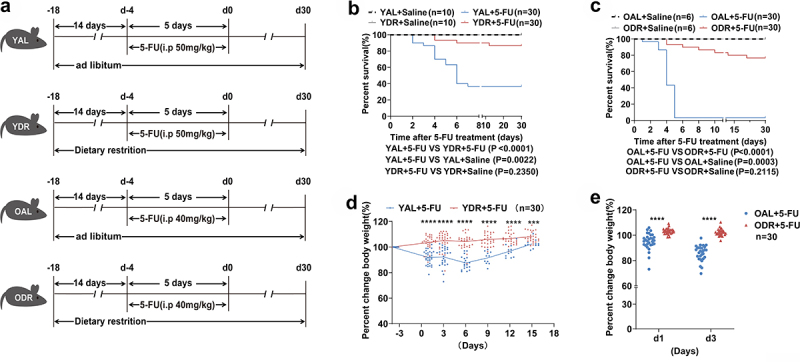
DR increases survival of 5-FU treated mice.

To investigate the effect of DR and the impact of aging on 5-FU treated mice, 2-month-old and 20 to 24-month-old mice (the equivalent change in humans would roughly be 67–80 years)^50^ were exposed to 30% DR or AL diet for 14 days before intraperitoneal 5-FU injection, which was performed daily for 5 days. A 20% lower dose of 5-FU was used in the old group to best account for their age and to see the protective effect by DR. For the control group, saline was injected (Figure 1a). Survival rates of mice were investigated in three independent experiments with a combined total of 30 mice per 5-FU group, 10 mice per saline group (young) and 6 mice per saline group (old). The results from the three experiments were combined for analysis due to the high level of consistency among all three independent experiments. Eleven young mice and one old mouse on AL diet survived after 5-FU treatment (survival rates: young AL mice 37%, old AL mice 3%), indicating a remarkably reduced survival rate in old mice. In young 5-FU treated AL mice, the survival rate was stable after day 7 until day 30, which marked the end of the monitoring period, while almost all old 5-FU treated AL mice died rapidly within 5 days. Intriguingly, survival rates were substantially increased the in DR groups, with 26 young mice (survival rate 87%) and 23 old mice (survival rate 76%) surviving after 5-FU treatment. In the DR groups, young mice died between day 4 and day 12, and old mice died between day 4 and day 21. No more mice died fter the end of the 30 days of monitoring period (Figure 1b,c). The bodyweight was also measured to further understand the overall health status. In line with the survival rates, AL mice showed a great loss of bodyweight after 5-FU treatment, while DR mice kept their bodyweights stable (Figure 1d,e). These results indicated that DR mice had remarkably better tolerance to 5-FU treatment than AL mice, which was more significant in old AL mice.

(A) Scheme of experiments. Young mice (2-month-old) and old mice (20–24-month-old) were exposed to AL diet or DR for 14 days before intraperitoneal 5-FU injection which was daily performed for 5 days, and the diet regimen was continued afterward. For the control group, saline was injected instead of 5-FU.

(B,C) Survival was monitored daily after 5-FU treatment (Data combined from 3 independent experiments. *n* = 30 mice per group for the AL + 5-FU group and DR + 5-FU group; *n* = 10 mice per group for the young AL+Saline group and young DR+Saline group; *n* = 6 mice per group for the old AL+Saline group and old DR+Saline group).

(D,E) Percent change of body weight at indicated timepoints after 5-FU treatment compared to before 5-FU treatment (*n* = 30 mice per group. Data combined from 3 independent experiments).

(B,C) Gehan-Breslow-Wilcoxon test; (D,E) Unpaired two-tailed, Student’s t test. Results were displayed as mean±SD. **p < .01; **** *p* < .0001. YAL+Saline: young mice on AL diet and received saline injection; YDR+Saline: young mice on DR diet and received saline injection; YAL + 5-FU: young mice on AL diet and received 5-FU injection; YDR + 5-FU: young mice on DR diet and received 5-FU injection. OAL+Saline: old mice on AL diet and received saline injection; ODR+Saline: old mice on DR diet and received saline injection; OAL + 5-FU: old mice on AL diet and received 5-FU injection; ODR + 5-FU: old mice on DR diet and received 5-FU injection; d1: day 1 after 5-FU injection; d3: day 3 after 5-FU injection.

### DR inhibits intestinal infection and opportunistic pathogens translocation

**Figure 2.**
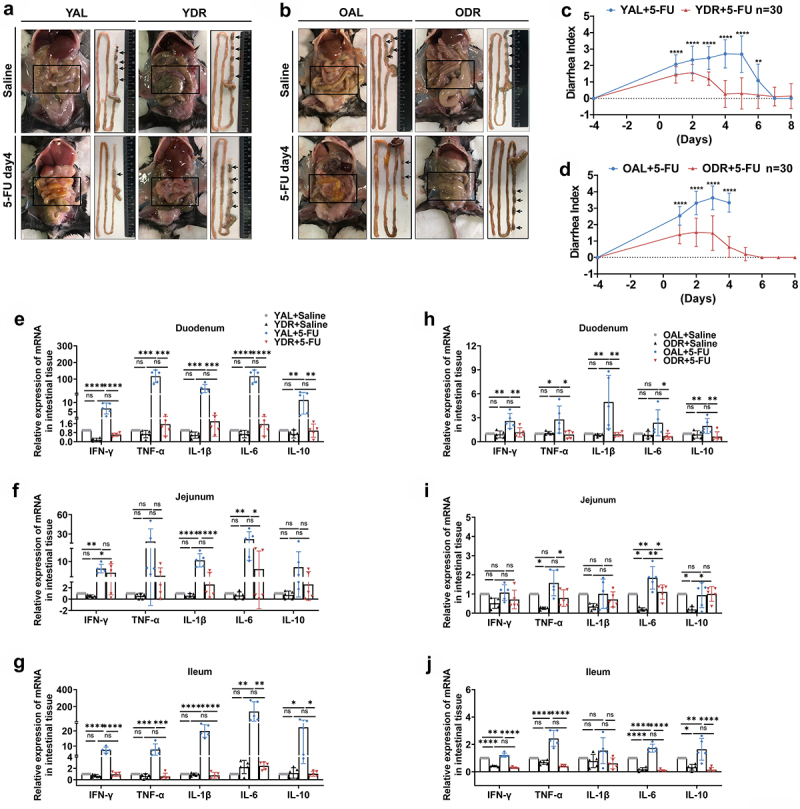
DR inhibits 5-FU-induced intestinal inflammation.

To investigate the reason behind the observed lethality of 5-FU treatment in AL mice and how DR rescued it, we first examined the intestines of mice on day 4 after 5-FU treatment because it was known that intestinal epithelium is a major target of 5-FU.^33,51,52^ Indeed, the intestines of AL mice exposed to 5-FU were covered with mucus and exhibited significant edema, multiple erosions, and hardly visible formed stools, while the intestines of DR mice treated with 5-FU looked similar to saline treated control mice with no visible damage in the intestinal wall and lots of formed stools (Figure 2a,b). Next, we scored diarrhea indexes by using the Bowen scoring system to further evaluate the intestinal toxicity by 5-FU. We found that the diarrhea score increased rapidly in AL mice with a peak on day 5 in young mice and a peak on day 3 in old mice after 5-FU treatment. The diarrhea score showed a milder increase in DR mice with a smaller peak on day 2 after 5-FU treatment, which quickly dropped down to a basal level as before 5-FU treatment (Figure 2c,d). We and others have shown previously that DR strongly inhibited inflammation under different conditions.^53,54^ As intestinal inflammation could be a major cause of diarrhea resulting from 5-FU toxicity to intestinal epithelial cells, we then examined the expression levels of inflammation-related genes (IFN-γ, TNF-α, IL-1β, IL-6, and IL-10) by quantitative PCR in duodenum, jejunum, and ileum tissues collected on day 3 after chemotherapy which is the time point of peak inflammation in old mice as indicated by diarrhea index. The results showed greatly elevated expression levels of the examined inflammatory genes throughout the intestine, especially TNF-α and IL-6 in AL mice after 5-FU treatment, while in DR mice, no significant change in the expression levels of these inflammatory genes was observed after 5-FU treatment in both young and old groups (Figure 2e–j). These results indicate that DR strongly inhibited intestinal inflammation and damage induced by 5-FU treatment. Of note, samples were collected on day 3 after chemotherapy for both young and old mice which was earlier than the time of peak inflammation in young mice. We speculate the difference between AL and DR mice could be bigger on day 5 after chemotherapy in young mice when the peak of inflammation took place in these groups.

Young mice (2-month-old) and old mice (20–24-month-old) were exposed to AL diet or DR for 14 days before intraperitoneal 5-FU injection which was daily performed for 5 days (day -4–day 0), and the diet regimen was continued afterward. For the control group, saline was injected instead of 5-FU. Mice were sacrificed at certain timepoints and small intestinal tissue was collected for further analysis.

(A,B) Representative photos of mice from indicated groups. Note that the intestines of AL mice exposed to 5-FU were covered with mucus and exhibited significant edema, multiple erosions, and hardly visible formed stools, while the intestines of DR mice treated with 5-FU looked similar to saline treated control mice with no visible damage in the intestinal wall and lots of formed stools (arrows).

(C,D) Bowen scoring system determined diarrhea score indexes from indicated groups (*n* = 30 mice per group. Data combined from three independent experiments).

(E-J) Relative expression of indicated genes in duodenum, jejunum, and ileum tissues collected on day 3 after 5-FU treatment by qRT-PCR (*n* = 5 mice per group randomly picked from 2 independent experiments).

(C,D) Unpaired two-tailed, Student’s t test; (E-J) One-way ANOVA test. Results were displayed as mean±SD. *p < .05; **p < .01;***p < .001; ****p < .0001; ns, not significant.

**Figure 3.**
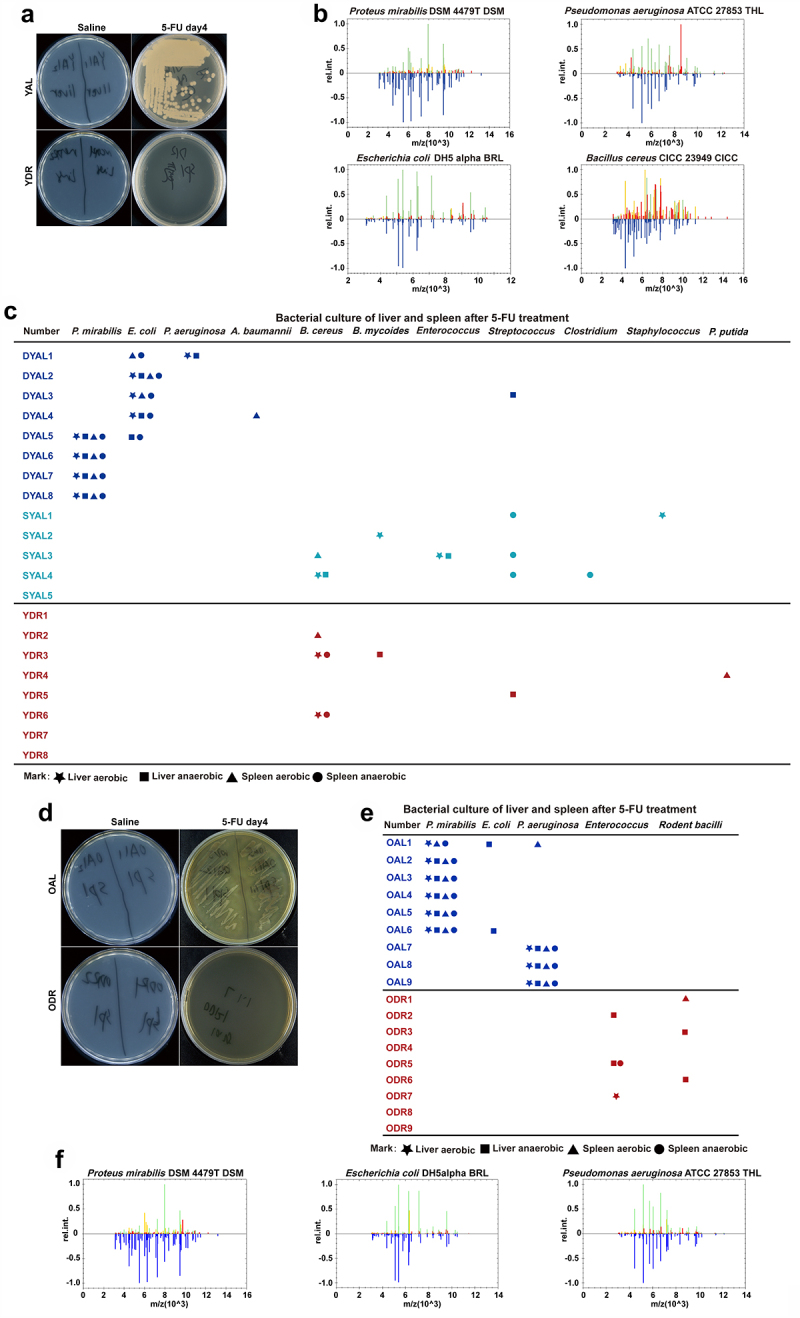
DR inhibits intestinal opportunistic pathogens translocation after 5-FU treatment.

It is known that the rapidly cycling intestinal epithelial cells are a target of 5-FU. We therefore hypothesized that 5-FU induced damage to intestinal barrier and translocation of intestinal bacteria, which then resulted in severe infection and death. To test this scenario, we first collected the liver and spleen on day 4 after 5-FU treatment and homogenized the tissue for bacterial culture and colony identification. Intriguingly, all the young mice and old mice which were not able to survive after 5-FU had opportunistic pathogens grown out from their liver and spleen homogenates. The identified opportunistic pathogens include *Proteus mirabilis*, *Escherichia coli, Pseudomonas aeruginosa*, and *Acinetobacter baumannii*, which are all highly virulent opportunistic pathogens and also common pathogens causing severe infections in clinical practices.^55–58^ *P.mirabilis* was the most prominently identified bacteria in both young non-survived mice and all old mice. *P.aeruginosa* was more frequently identified in old mice. On the contrary, none of the survived mice had such opportunistic pathogens grown out from their liver and spleen homogenates. One out of five survived AL mice, with five out of eight young DR mice and six out of nine old DR mice possessing opportunistic pathogens with low virulence.^59–61^ These results presented clear evidence that 5-FU treatment leads to penetration of opportunistic pathogens with highly virulence through the intestinal barrier, allowing for their translocation to distant organs, which dominantly takes place in old mice and could be completely blocked by DR (Figure 3a–f).

Young mice and old mice (20–24-month-old) were exposed to AL diet or DR for 14 days before intraperitoneal 5-FU injection which was daily performed for 5 days (day -4–day 0), and the diet regimen was continued afterward. For the control group, saline was injected instead of 5-FU. Mice were sacrificed on day 4 after 5-FU treatment and liver and spleen were collected and homogenized for bacterial culture and further analysis.

(A,D) Representative pictures of bacteria culture dishes from indicated groups.

(B,F) The bacterial colonies grown out from the liver and spleen homogenates were identified by mass spectrometer. Above the ordinate 0 scale is the key mass spectral peak data of the target bacteria, which is compared with the known strain spectrum in the database located below the ordinate 0 scale identifying specific bacteria.

(C,E) Bacterial species identified from colonies grown out from individual mouse tissue homogenates. (Data combined from two independent experiments). Mice which met Death or Moribundity Criteria according to Guidelines for Endpoints in Animal Study Proposals after 5-FU were defined as non-survived or dead. Mice which did not meet Death or Moribundity Criteria were defined as survived. DYAL: dead young *ad libitum*; SYAL: survived young *ad libitum*.

**Figure 4.**
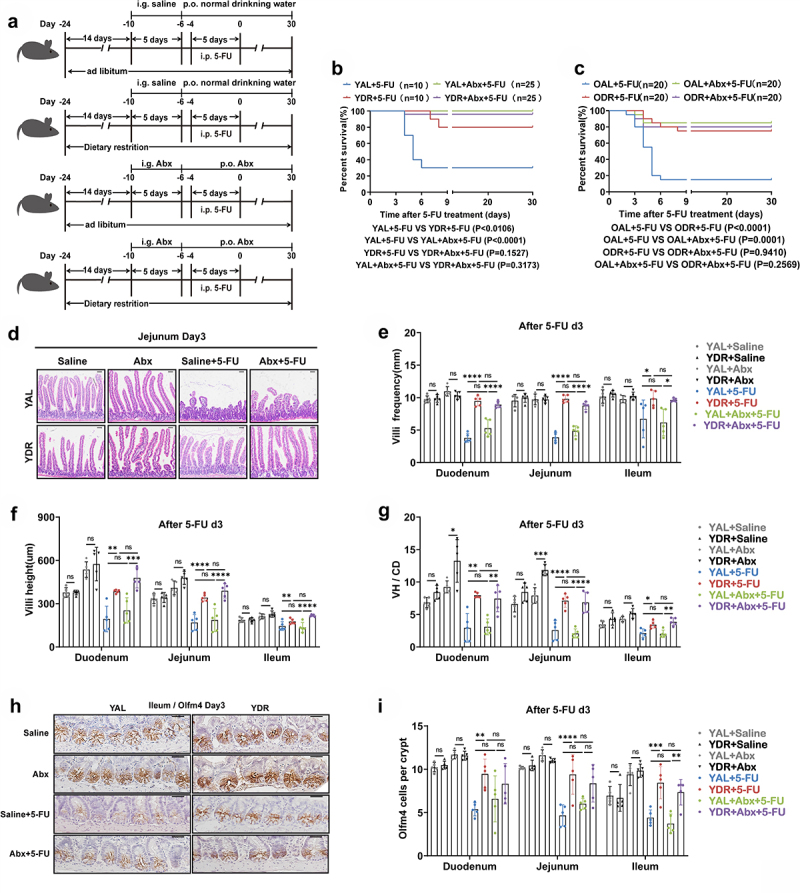
Abx administration rescued survival of AL mice after 5-FU treatment.

To further examine whether 5-FU-induced translocation of intestinal bacteria allowed for infection and death, we then administrated broad-spectrum antibiotics (Abx) prior to 5-FU treatment with the purpose of clearing gut microbiota and investigated the survival rates (Figure 4a). Notably, the low survival rate of AL mice was significantly rescued by the Abx administration in both young and old mice (Figure 4b,c). To further examine whether the rescue resulted from protection of intestinal epithelium or simply wiping out the intestinal bacteria, we checked the histology of small intestinal tissue by H&E staining and found no significant change in villi frequencies, villi heights, and the ratio of villi height versus crypt depth (VH/CD) by Abx administration, indicating neutral effect of Abx on intestinal epithelial damage (Figure 4d–g). The immunohistochemical staining of Olfm4, an intestinal stem cell (ISC) marker, also showed no protecting effect on ISCs by Abx (Figure 4h–i). Since the rescue of survival by Abx was similar in young and old mice, histological analysis was performed only in young mice to save the use of old mice. Together, these results indicated that infection from translocated intestinal pathogenic bacteria may be a major cause of death to mice receiving 5-FU treatment.

(A) Scheme of experiments. Young mice (2-month-old) and old mice (20–24-month-old) were exposed to AL diet or DR for 14 days before intraperitoneal 5-FU injection which was daily performed for 5 days (day-4–day0), and the diet regimen was continued afterward. Seven days (day-10) prior to 5-FU administration, mice were given a gavage of broad-spectrum antibiotics (Abx group) or saline (control group) for 5 days and then in drinking water for the following days.

(B,C) Survival was monitored daily after 5-FU treatment (Data combined from 2 independent experiments. *n* = 10 mice per group for the young AL + 5-FU group and DR + 5-FU group; *n* = 25 mice per group for the young AL+Abx +5-FU group and DR+Abx +5-FU group; *n* = 20 mice per group for the old AL + 5-FU group, DR + 5-FU group, AL+Abx +5-FU group, and DR+Abx +5-FU group).

(D) Representative images of H&E staining of jejunum on day 3 after 5-FU treatment from indicated groups. Scale bar: 50 μm.

(E-G) Villi frequencies, villi height, and VH/CD (the ratio of villi height versus crypt depth) (*n* = 5 mice per group randomly picked from 2 independent experiments).

(H) Representative images of Immunohistochemistry staining of Olfm4 of ileum on day 3 after 5-FU treatment from indicated groups. Scale bar: 20 μm.

(I) Basal crypt Olfm4-positive cell number per crypt on day 3 after 5-FU treatment (*n* = 5 mice per group randomly picked from 2 independent experiments).

(B,C) Gehan-Breslow-Wilcoxon test; (E-G, I) One-way ANOVA test. Results were displayed as mean±SD. *p < .05; **p < .01;***p < .001; ****p < 0.0001; ns, not significant. YAL+Saline: young mice on AL diet and received saline injection; YDR+Saline: young mice on DR diet and received saline injection; YAL + 5-FU: young mice on AL diet and received 5-FU injection; YDR + 5-FU: young mice on DR diet and received 5-FU injection;YAL+Abx +5-FU: young mice on AL diet and received a gavage of Abx and 5-FU injection; YDR+Abx +5-FU: young mice on DR diet and received a gavage of Abx and 5-FU injection. d3: day3 after 5-FU injection; Abx: broad-spectrum antibiotics.

### DR significantly reduced the opportunistic pathogens in the gut of mice exposed to 5-FU

**Figure 5.**
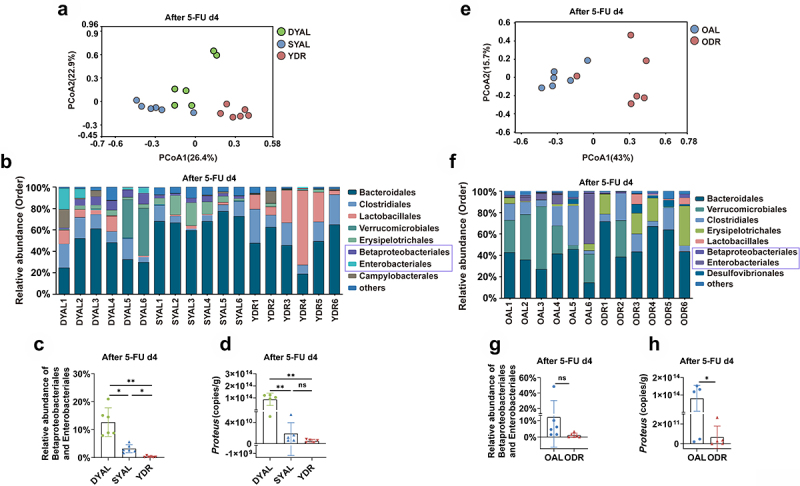
DR reduced the opportunistic pathogens in the gut microbiota of mice after 5-FU treatment.

Given a remarkable difference in the translocation of intestinal opportunistic pathogens between the non-survived mice and survived mice after 5-FU treatment, we first asked whether the amount of opportunistic pathogens in the gut was different among these mice. To answer this question, fecal samples were collected on day 4 after 5-FU treatment for gut microbiota analysis by 16S rRNA gene deep-sequencing (Illumina 250 bp paired-end) and quantitative PCR. This was a time point before the start of massive death and heavy diarrhea hampered the collection of feces in the AL group. The principal-coordinate analysis (PCoA) based on Bray-Curtis distance showed that the overall structure of the gut microbiota was significantly changed by DR in young mice (Figure 5a). Furthermore, analysis on the composition of the gut microbiota on the order level showed a higher percentage of opportunistic pathogens (including Betaproteobacterales and Enterobacteriales) in young AL mice (Figure 5b). Further statistical analysis showed that the relative abundance of Betaproteobacteriales and Enterobacteriales in the gut microbiota of young DR mice was almost undetectable, while it was increased in a small range in the survived young AL mice, and was remarkably increased in the non-survived young AL mice (Figure 5c). To further determine the difference of opportunistic pathogens in the gut, quantitative PCR on *Proteus* was performed. Astonishingly, the abundance of *Proteus* in the feces of non-survived young AL mice was 8.83 ± 5.10 × 10^13^ copies/g, which was around ten thousand fold higher than the survived AL mice (1.91 ± 2.03 × 10^10^ copies/g), and twenty thousand fold higher than the DR mice (4.97 ± 2.87 × 10^9^ copies/g) (Figure 5c,d). The same experiments were performed on old mice and a similar pattern of bacteria change was observed, with a greatly increased amount of opportunistic pathogens detected in the old AL mice (Figure 5e–h). In particular, quantitative PCR showed that the abundance of *Proteus* in old AL mice was one thousand fold higher than old DR mice (8.06 ± 7.45 × 10^13^ copies/g in old AL mice versus 6.65 ± 1.13 × 10^11^ copies/g in old DR mice). These results indicated that the amount of the opportunistic pathogens in the intestines of those non-survived AL mice was much greater when compared to DR mice, which in turn could increase the probability for the penetration of these bacteria through the intestinal barrier and their subsequent translocation.

Young mice (2-month-old) and old mice (20–24-month-old) were exposed to AL diet or DR for 14 days before intraperitoneal 5-FU injection which was daily performed for 5 days (day -4–day 0), and the diet regimen was continued afterward. Fecal samples were randomly collected from mice on day 4 after 5-FU injection for 16S rRNA gene sequencing (*n* = 6 mice per group randomly picked from 2 independent experiments) and qPCR analysis (*n* = 5 mice per group randomly picked from 2 independent experiments).

(A,E) Variation of intestinal microbiota structure of indicated groups along PC1 and PC2 of PCoA based on the Bray – Curtis distance.

(B,F) Relative abundance of the intestinal microbiota of indicated groups on the order level showed by 16S rRNA gene sequencing.

(C,J) Relative abundance of the Betaproteobacterales and Enterobacteriales showed by 16S rRNA gene sequencing.

(D,H) qPCR analysis of the amounts of *Proteus* of indicated groups.

(C,D) One-way ANOVA test;(G,H) Unpaired two-tailed,Student’s t test; Results were displayed as mean±SD; ns, not significant; * *p* < .05; **p < 01. Mice which met Death or Moribundity Criteria according to Guidelines for Endpoints in Animal Study Proposals after 5-FU were defined as non-survived or dead. Mice which did not met Death or Moribundity Criteria were defined as survived. DYAL: dead young *ad libitum*; SYAL: survived young *ad libitum*.

### DR significantly protects intestinal physical barrier from 5-FU

**Figure 6.**
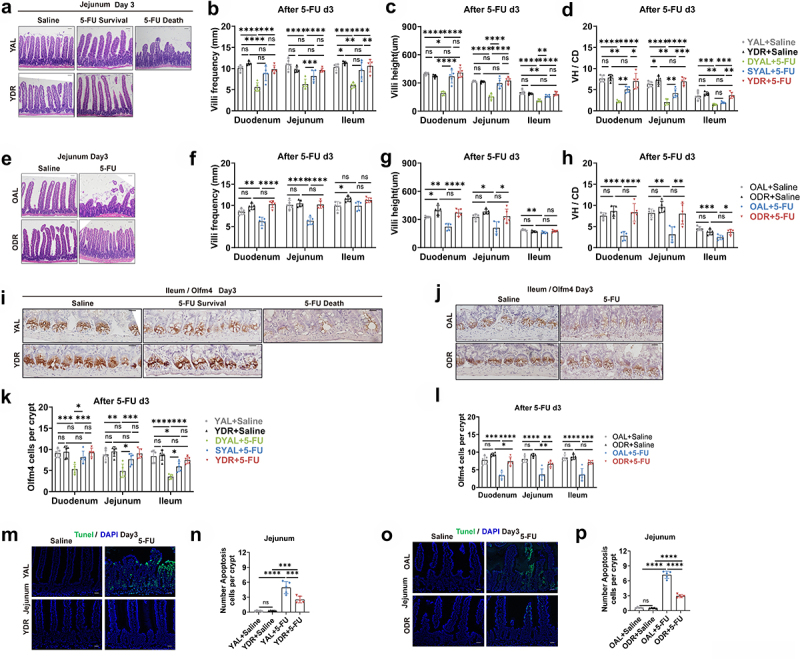
DR protects intestinal physical barrier from 5-FU.

The intestinal barrier is primarily composed of a physical barrier, a biological barrier, and a chemical barrier, which all work harmoniously to block the translocation of intestinal bacteria.^62,63^ Given the remarkable difference in the translocation of intestinal opportunistic pathogens between the AL and DR mice after 5-FU treatment, we asked whether DR regulated the intestinal barrier to protect the 5-FU toxicity. To answer this question, we first examined the physical barrier by histological and immunohistochemistry staining. Notably, H&E staining showed that 5-FU treatment induced significant damage to the small intestine of non-survived AL mice, including reduced villi frequencies, villi heights, and the ratio of VH/CD through different fractions of the small intestine (VH/CD) (Figure 6a–h). Interestingly, in both young and old groups, DR maintained villi heights, frequencies and the ratio of VH/CD in mice exposed to the same level of 5-FU, indicating a significant protection of intestinal epithelium achieved by DR (Figure 6a–h).

Young mice (2-month-old) and old mice (20–24-month-old) were exposed to AL diet or DR for 14 days before intraperitoneal 5-FU injection which was daily performed for 5 days (day -4–day 0), and the diet regimen was continued afterward. For the control group, saline was injected instead of 5-FU. The small intestines were collected on day3 after 5-FU treatment for further analysis (*n* = 5 mice per group randomly picked from 2 independent experiments).

(A,E) Representative images of H&E straining of ileum from indicated groups. Scale bar: 50 μm.

(B-H) Villi frequencies, villi height, and VH/CD (the ratio of villi height versus crypt depth) (*n* = 5 mice per group randomly picked from 2 independent experiments).

(I,J) Representative images of Immunohistochemistry staining of Olfm4 of ileum. Scale bar: 20 μm.

(K,L) Basal crypt Olfm4-positive cell number per crypt in indicated fractions of the intestine from indicated groups (30 crypts were counted for each fraction. *n* = 5 mice per group randomly picked from 2 independent experiments).

(M,O) Representative images of TUNEL staining of jejunum. Scale bar: 20 μm.

(N,P) Crypt apoptotic cell number per crypt in jejunum as determined by TUNEL staining.

(B-D,F-H,K,L,N,P) One-way ANOVA test. Results were displayed as mean±SD. *p < .05; **p < .01;***p < .001; ****p < .0001; ns, not significant. Mice which met Death or Moribundity Criteria according to Guidelines for Endpoints in Animal Study Proposals after 5-FU were defined as non-survived or dead. Mice which did not met Death or Moribundity Criteria were defined as survived. DYAL: dead young *ad libitum*; SYAL: survived young *ad libitum*.

To examine the effect of DR on small intestinal stem cells, IHC staining for Olfm4 was performed on small intestinal sections. In young mice, 5-FU induced a significant loss of Olfm4-positive ISCs in the non-survived AL mice, while no obvious loss of ISCs was observed in DR mice and the survived AL mice, except for a mild reduction in the ileum of the non-survived AL mice (Figure 6i,k). In the old mice, the loss of Olfm4+ ISCs was more significant compared to the young mice, and even DR mice showed slightly reduced ISC number in duodenum and jejunum but to a much smaller extent comparing to the AL mice (Figure 6j,l). To further explore the reason of villi shortening and loss of ISCs, TUNEL staining was performed to visualize apoptosis (Figure 6m,o). In both young and old mice, 5-FU treatment led to a clear increase of the number of apoptosis cells, but the degree of increase in DR mice was much less comparing to the AL mice. Moreover, old AL mice showed a higher number of apoptotic cells per crypt when compared to young mice (7.21 in OAL vs 4.95 in YAL), while the numbers of apoptotic cells of young DR mice and old DR mice were similar (2.938 in ODR vs 2.522 in YDR) (Figure 6n,p). The intact intestinal epithelium preserved by DR could therefore serve as a better physical barrier to limit the access of enteric microbes and reduce the induction of inflammation response induced by 5-FU.

### DR significantly protects intestinal biological barrier from 5-FU

**Figure 7.**
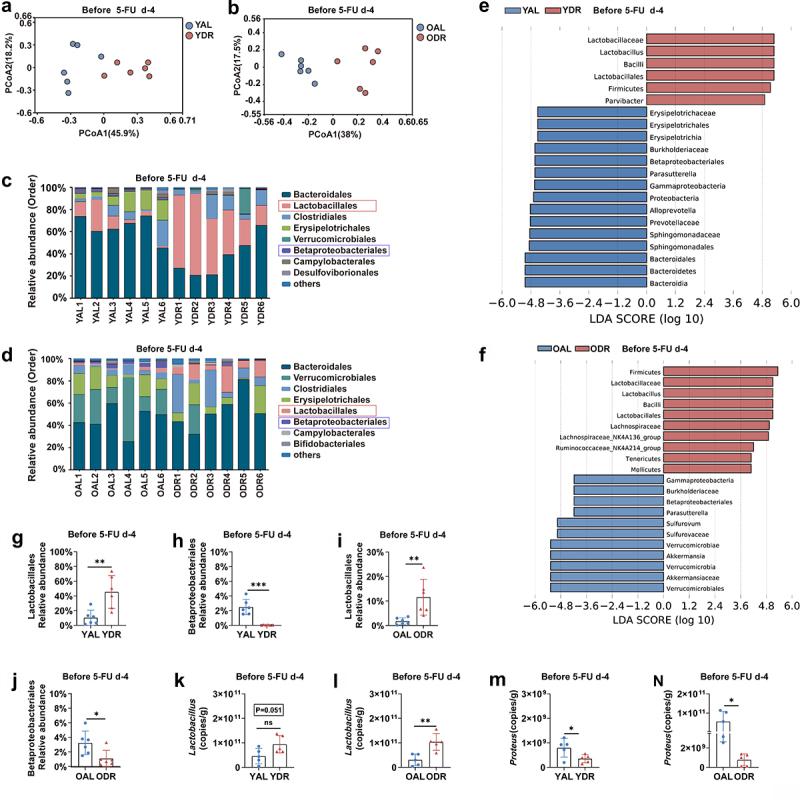
DR protects intestinal biological barrier from 5-FU.

Intestinal commensal microbes are known to provide a biological barrier to maintain intestinal homeostasis and health via direct and indirect mechanisms.^64^ To investigate the impact of DR on the intestinal biological barrier, feces were collected from mice after two weeks of AL or DR diet before 5-FU treatment and the gut microbiota were analyzed by 16S rRNA gene deep-sequencing (Illumina 250 bp paired-end). Interestingly, principal-coordinate analysis (PCoA) based on Bray-Curtis distance showed that the short-term DR before 5-FU treatment already led to a significant shift of the overall structure of the gut microbiota in both young and old mice (Figure 7a,b). Further analysis showed that the composition of the gut microbiota at the level of order was significantly shifted with a most prominently relative abundance increase of the order Lactobacillales and decrease of the order Betaproteobacteriales in DR mice (Figure 7c,d). We then used the linear discriminant analysis (LDA) scores over 4 and looked at the top 15 bacteria in each direction. The order of Lactobacillales, family of Lactobacillaceae and genus of *Lactobacillus* were among the top 5 enriched taxa in young DR mice comparing to young AL mice (Figure 7e). When comparing old DR mice to old AL mice, the order of Lactobacillales, family of Lactobacillaceae and genus of *Lactobacillus* were also among the top 5 enriched taxa (Figure 7f). Statistical analysis further indicated significant increases of Lactobacillales and decreases of Betaproteobacteriales in their relative abundance in the gut microbiota by DR treatment in both young and old mice (Figure 7g–j). To further determine the quantitative change of *Lactobacillus* and *Proteus*, quantitative PCR (qPCR) was performed. The results show a trend of increase in the copy number of *Lactobacillus* per gram feces in young DR mice (4.57 ± 3.13 × 10^10^ copies/g versus 9.44 ± 3.58 × 10^10^ copies/g, *p* = .051) (Figure 7k). In old AL mice, the absolute mean value of the copy number of *Lactobacillus* per gram feces was less compared to young AL mice (3.04 ± 2.33 × 10^10^ copies/g versus 4.57 ± 3.13 × 10^10^ copies/g), and it was significantly increased after the short-term exposure to DR which was similar level to young DR mice (1.04 ± 3.46 × 10^10^ copies/g in old DR mice, 9.44 ± 3.58 × 10^10^ copies/g in young DR mice) (Figure 7l). *Lactobacillus* is a normal intestinal bacteria taxa which is closely integrated with the intestinal mucosa and constitutes part of the biological barrier preventing the invasion of pathogenic bacteria. The increased amount of *Lactobacillus* in DR mice could serve as a strengthened biological barrier that inhibits the growth of pathogenic bacteria and their invasion into blood. Indeed, quantitative analysis showed that the amount of *Proteus* was already reduced by the short-term exposure to DR before 5-FU treatment in both young and old mice (Figure 7m,n). Notably, the mean value of absolute amount of *Proteus* in old AL mice was more than one hundred fold higher than in the young AL mice (5.58 ± 5.32 × 10^10^ in old AL mice versus 7.98 ± 3.78 × 10^8^ in young AL mice), while it was similar in old and young DR mice (7.79 ± 6.14 × 10^8^ in old DR mice, and 3.54 ± 1.68 × 10^8^ in young DR mice) (Figure 7m,n). Astonishingly, when we compare the absolute amount of *Proteus* after 5-FU treatment to before 5-FU treatment, we observed a one-thousand fold increase in old AL mice (8.06 ± 7.45 × 10^13^ versus 5.58 ± 5.32 × 10,^10^ whereas it was only one-hundred-fold increased in old DR mice (6.65 ± 1.13 × 10^11^ versus 7.79 ± 6.14 × 10^8^ (Figures 5h and 7n). The results indicated that the growth of pathogenic bacteria was tremendously inhibited by DR, and that a microbial disturbance took place after 5-FU treatment, which was significantly rescued by DR.

Fecal samples were randomly collected from in young and old mice after two weeks of AL or DR diet before 5-FU treatment and gut microbiota were analyzed by 16S rRNA gene sequencing (*n* = 6 mice per group randomly picked from 2 independent experiments) and qPCR analysis (*n* = 5 mice per group randomly picked from 2 independent experiments).

(A,B) Principal-coordinate analysis (PCoA) based on Bray-Curtis distance showed a significant shift of the overall structure of the gut microbiota by DR in both young and old mice.

(C,D) Relative abundance of the intestinal microbiota of indicated groups on the order level showed by 16S rRNA gene sequencing.

(E,F) LDA scores in the fecal microbiomes of indicated groups. LDA score > 4 and top fifteen bacteria were shown.

(G-J) Relative abundance of the Lactobacillales and Betaproteobacteriales showed by 16S rRNA gene sequencing.

(K-N) qPCR analysis of the amounts of *Lactobacillus* and *Proteus* of indicated groups.

(G-N) Unpaired two-tailed, Student’s t test. Results were displayed as mean±SD. *p < .05; **p < .01;***p < .001; ns, not significant.

### LGG gavage partially rescues survival of AL mice exposed to 5-FU

**Figure 8.**
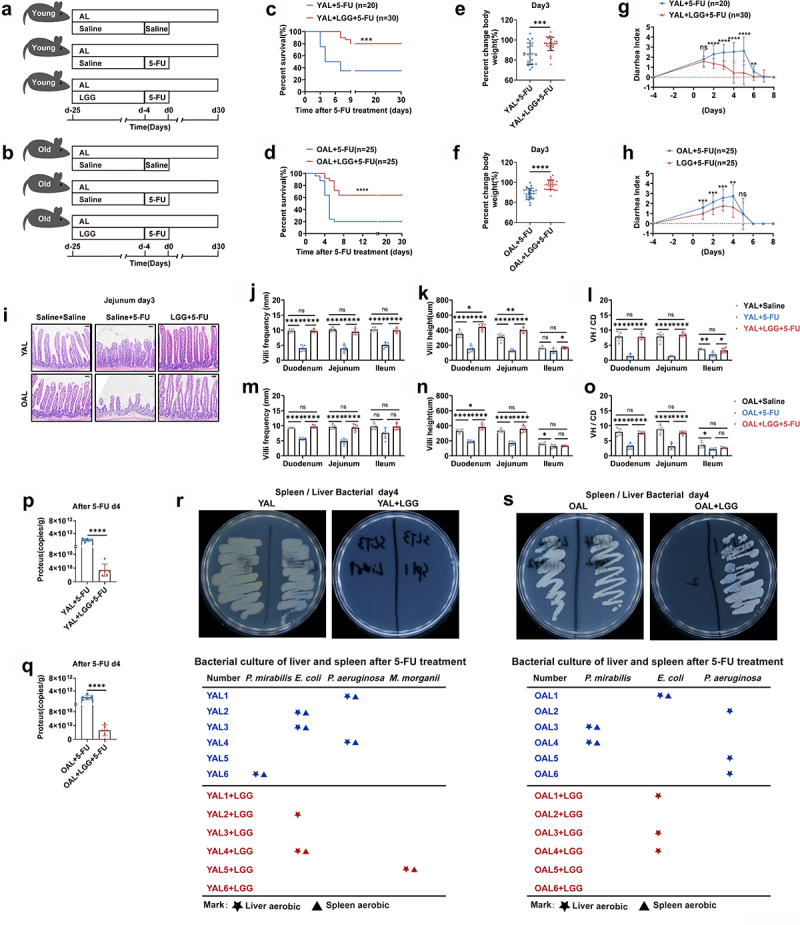
LGG gavage partially rescues survival of AL mice exposed to 5-FU.

The above results suggested that an increase in *Lactobacillus* after DR may play a role in protecting the intestinal barrier upon 5-FU insult. To investigate this, we gavaged AL mice with LGG which is known to protect against chemotherapy-induced intestinal toxicities.^23,65,66^ LGG was applied to AL mice continuously for 3 weeks before 5-FU treatment (Figure 8a,b). Interestingly, LGG increased the survival rate to 80% in young AL mice and 64% in old AL mice by day 9 after 5-FU treatment, and the survival rate was stable until the end of the monitoring period on day 30 (Figure 8c,d). In line with that, LGG resulted in a better maintenance of bodyweight (Figure 8e,f). The diarrhea index was also significantly reduced, indicating less severe diarrhea by LGG administration (Figure 8g,h). Furthermore, the histological analysis on small intestine showed that LGG gavage significantly improved maintenance of villi frequencies, villi height and the ratio of VH/CD, indicating that the small intestinal architecture was protected from 5-FU treatment by LGG gavage (Figure 8i–o).

(A,B) Scheme of experiments. Young mice (2-month-old) and old mice (20–24-month-old) were treated with LGG or saline by gastric gavage for 3 weeks before 5-FU treatment (1 × 10^9^ CFU LGG per mouse per day). Then intraperitoneal 5-FU or saline (control) injection was daily performed for 5 days(day -4,day 0). In the young group: data were combined from 3 independent experiments (*n* = 20 mice per group for the AL + 5-FU group, *n* = 30 mice per group for the AL+LGG +5-FU group). In the old group: data were combined from 3 independent experiments. (*n* = 25 mice per group for the AL + 5-FU group and the AL+LGG +5-FU group).

(C,D) Survival was monitored daily after 5-FU treatment.

(E,F) Percent change of bodyweight on day 3 after 5-FU treatment.

(G,H) Bowen scoring system determined diarrhea score indexes from indicated groups.

(I-O) Small intestines were collected for further analysis. (I) Representative images of H&E straining of Jejunum on day 3 after 5-FU treatment. Scale bar: 50 μm. (J-O) Villi frequencies, villi height, and VH/CD (the ratio of villi height versus crypt depth) (*n* = 5 mice per group randomly picked from 2 independent experiments).

(P,Q) Mice were sacrificed on day 4 after 5-FU treatment and liver and spleen were collected and homogenized for bacterial culture and further analysis. Representative pictures of bacteria culture dishes from indicated groups were shown. The bacterial colonies grown out from the liver and spleen homogenates were identified by mass spectrometer. The bellowing tables show bacterial species identified from colonies grown out from individual mouse tissue homogenates (*n* = 6 mice per group from 2 independent experiments).

(R,S) Fecal samples were randomly collected on day 4 after 5-FU treatment. The amounts of *Proteus* were determined by qPCR analysis of (*n* = 6 mice per group from 2 independent experiments).

(C,D) Gehan-Breslow-Wilcoxon test; (E-H,P,Q) Unpaired two-tailed, Student’s t test; (J-O) One-way ANOVA test. Results were displayed as mean±SD. *p < .05; **p < .01;***p < .001; ****p < 0.0001; ns, not significant.

Being one of the prominent bacteria taxa belonging to the *Lactobacillus* genus, increased amount of LGG could function as an important biological barrier by being in close proximity to the intestinal mucosa to prevent invasion of pathogenic bacteria. To examine this scenario, qPCR was performed to determine the quantitative change of *Proteus* in the intestines of AL mice in response to LGG gavage. Indeed, LGG gavage led to a significant reduction of the abundance of *Proteus* in both young and old mice (Figure 8p,q). Next, we performed bacterial culture and colony identification from homogenized liver and spleen tissue. LGG gavage reduced number of mice exhibiting translocation of intestinal opportunistic pathogens in both young and old AL groups (Figure 8r,s). This result indicated that LGG gavage suppressed the growth of opportunistic pathogens in the intestines and inhibited their translocation to distant organs after 5-FU treatment, which further supports the scenario that the modulated structure of gut microbiota by DR improved the biological barrier and protected mice from 5-FU.

### DR significantly protects intestinal chemical barrier from 5-FU

**Figure 9.**
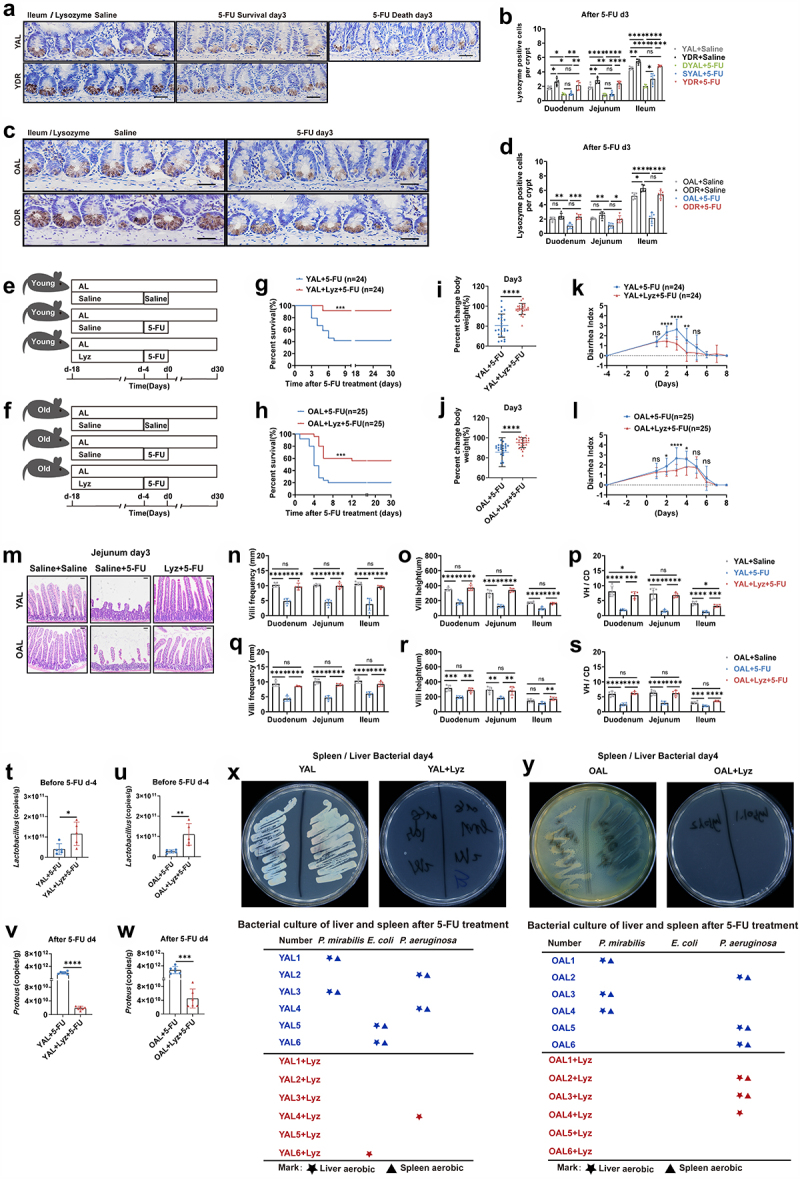
DR protects intestinal chemical barrier from 5-FU and lysozyme gavage partially rescues survival of AL mice exposed to 5-FU.

Paneth cells located at the bottom of the intestinal crypts produce lysozyme, an enzyme that can regulate the intestinal microbiota. It has been shown that *Lactobacillus* are resistant to lysozyme, whereas opportunistic pathogens are sensitive.^67–70^ Therefore, lysozyme serves as an important player in the chemical barrier of intestines. Intriguingly, short-term DR before 5-FU treatment already led to a significant increase of lysozyme-positive cells per crypt throughout different fractions of the small intestine in young mice (Figure 9a–d). In old mice more lysozyme-positive cells were seen in the duodenum and jejunum, with a significant increase in the ileum, which is the fraction that harbor largest amount of Paneth cells (Figure 9b,d).^71^ 5-FU treatment resulted in a significant loss of lysozyme-positive cells in both young and old AL mice, while the number of lysozyme-positive cells did not significantly change in DR mice (Figure 9a–d). The extent of increase of the amount of lysozyme-positive cells in DR mice comparing to AL mice was greater after 5-FU treatment than before the treatment (Figure 9a–d). We also observed a significantly higher number of lysozyme-positive cells in the ileum of survived AL mice than non-survived AL mice after 5-FU treatment (Figure 9b,d). This result further indicated that the increased amount of lysozyme-positive cells (especially after 5-FU treatment) might play an important role in survival of mice exposed to 5-FU. To further test the hypothesis, young and old AL mice were administrated with lysozyme by gastric gavage for 2 weeks before 5-FU treatment (Figure 9e,f). Intriguingly, lysozyme gavage achieved a significant increase of survival rate and better maintenance of bodyweight after 5-FU treatment in both young and old mice (Figure 9g–j). The diarrhea index was also reduced by lysozyme gavage before 5-FU treatment (Figure 9k,l). In line with that, the histological structure of the small intestine was protected from severe damage by 5-FU treatment via lysozyme gavage as shown by significantly maintained villi frequencies, villi height, and the ratio of VH/CD (Figure 9m–s).

(A-D)Young mice (2-month-old) and old mice (20–24-month-old) were exposed to AL diet or DR for 14 days before intraperitoneal 5-FU injection which was daily performed for 5 days, and the diet regimen was continued afterward. For the control group, saline was injected instead of 5-FU. Small intestinal tissue were collected on day 3 after 5-FU treatment for immunohistochemistry staining of Lysozyme. (A,C) Representative images of ileum. Scale bar: 20 μm. (B,D) Statistic analysis of number of Lysozyme-positive cells per crypt in indicated fractions of indicated groups (*n* = 5 mice per group randomly picked from 2 independent experiments).

(E,F) Scheme of experiments. (E) Young mice (2-month-old) and (F) old mice (20–24-month-old) were treated with lysozyme by gastric gavage for 2 weeks before 5-FU treatment. Then, intraperitoneal 5-FU or saline (control) injection was daily performed for 5 days (day -4,day 0). In the young group: data were combined from 3 independent experiments (*n* = 24 mice per group for the AL + 5-FU group and AL+Lyz +5-FU group). In the old group: data were combined from 3 independent expriments (*n* = 25 mice per group for the AL + 5-FU group and AL+Lyz +5-FU group). And the intestines were collected for further analysis (*n* = 5 mice per group randomly picked from 2 independent experiments).

(G,H) Survival was monitored daily after 5-FU treatment.

(I,J) Percent change of body weight on day 3 after 5-FU treatment.

(K,L) Bowen scoring system determined diarrhea score indexes from indicated groups.

(M-S) Small intestines were collected for further analysis. (M) Representative images of H&E straining of Jejunum on day 3 after 5-FU treatment. Scale bar: 50 μm. (N-S) Villi frequencies, villi height, and VH/CD (the ratio of villi height versus crypt depth) (*n* = 5 mice per group randomly picked from 2 independent experiments).

(P,Q) Mice were sacrificed on day4 after 5-FU treatment and liver and spleen were collected and homogenized for bacterial culture and further analysis. Representative pictures of bacteria culture dishes from indicated groups were shown. The bacterial colonies grown out from the liver and spleen homogenates were identified by mass spectrometer. The bellowing tables show bacterial species identified from colonies grown out from individual mouse tissue homogenates (*n* = 6 mice per group from 2 independent experiments).

(T-W) Fecal samples were randomly collected before (day -4) and after (day 4) 5-FU treatment. The amounts of *Lactobacillus* and *Proteus* were determined by qPCR analysis of (*n* = 6 mice per group from 2 independent experiments).

(G,H) Gehan-Breslow-Wilcoxon test; (I-L,T-W) Unpaired two-tailed, Student’s t test; (B,D,N-S) One-way ANOVA test. Results were displayed as mean±SD. *p < .05; **p < .01;***p < .001; ****p < .0001; ns, not significant.

Interestingly, further qPCR analysis revealed that lysozyme administration significantly increased the abundance of *Lactobacillus* in the intestines before 5-FU and decreased *Proteus* after 5-FU treatment (Figure 9t–w). Moreover, bacterial culture of homogenized liver and spleen from mice after 5-FU treatment showed that lysozyme gavage resulted in reduced number of mice having opportunistic pathogens grown out in both young and old group (Figure 9x,y). These results further indicate that 5-FU treatment resulted in significantly diminished lysozyme in the intestine of AL mice, especially in old ones, which led to increased translocation of intestinal opportunistic pathogens, could be an important mechanism of the increased intestinal toxicity and death observed in old mice.

### Relative abundance of opportunistic pathogens increases after 5-FU chemotherapy in old patients

**Figure 10.**
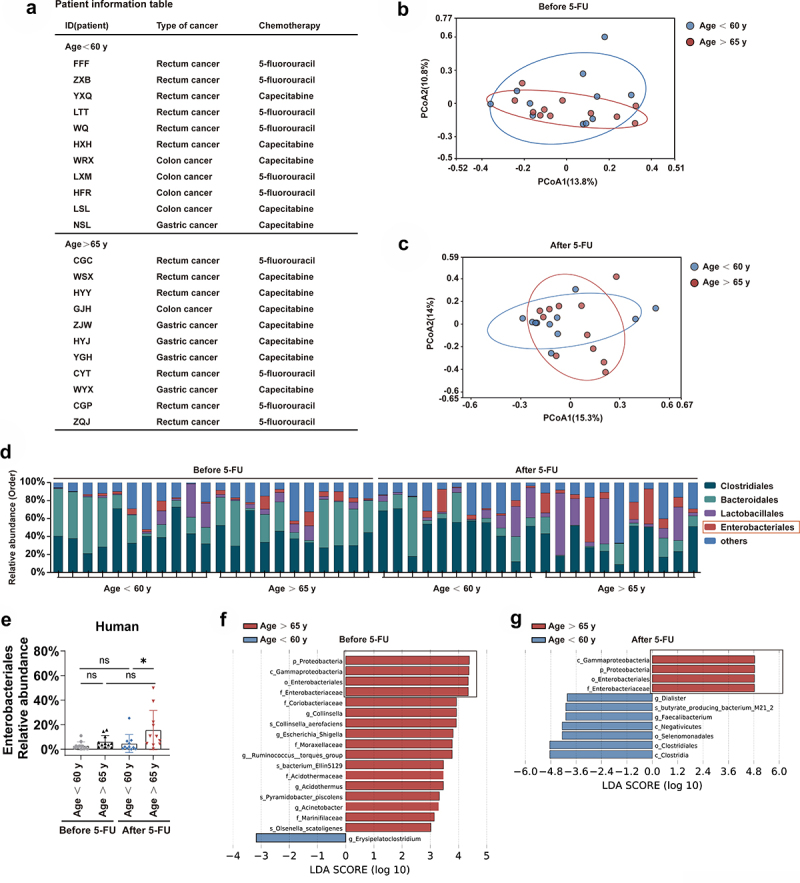
Relative abundance of opportunistic pathogens increases after 5-FU chemotherapy in old patients.

To further investigate the potential relevance and mechanism underlying the increased intolerability to chemotherapy in elderly patients, we collected feces from clinical patients before and after receiving 5-FU-based chemotherapy (Figure 10a). 16S rRNA gene deep-sequencing shows that the overall structure of patients was slightly different between patients less than 60 years old and patients older than 65 years before chemotherapy, and the difference seemed to be more apparent after chemotherapy (Figure 10b,c). Interestingly, older patients have a trend of increased relative abundance of the opportunistic pathogens Enterobacteriales than the younger ones, which became significant after chemotherapy (Figure 10d,e). Furthermore, we used the linear discriminant analysis (LDA) scores over 3 and looked at the top 15 bacteria in each direction. Intriguingly, opportunistic pathogens, including p_Proteobacteria, c_Gammaproteobacteria, o_Enterobacteriales and f_Enterobacteriaceae, were the top 4 enriched taxa in older patients comparing to younger ones before chemotherapy (Figure 10f). These pathogenic bacteria remain the top 4 enriched bacteria taxa in the older patients group after chemotherapy (Figure 10g).

Fecal samples of patients with gastrointestinal cancers were collected for 16S rRNA gene sequencing before the first cycle of 5-FU chemotherapy and one week/two weeks after the 5-FU treatment (*n* = 11 patients in the group aging less than 60 years old, and *n* = 11 patients in the group aging over 65 years old).

Patient information.

(B,C) Variation of intestinal microbiota structure of indicated groups along PC1 and PC2 of PCoA based on the Bray – Curtis distance.

(D) Relative abundance of the intestinal microbiota of indicated groups on the order level showed by 16S rRNA gene sequencing.

(E) Relative abundance of the Enterobacteriales showed by 16S rRNA gene sequencing.

(F,G) LDA scores in the fecal microbiomes of indicated groups. LDA score > 3 and top seventeen bacteria were shown.

(E) One-way ANOVA test, Results were displayed as mean±SD; *p < .05; ns, not significant.

## Discussion

Our study clearly indicates that even with a reduced dose of 5-FU, the survival rate of old mice is considerably lower than that of young mice after 5-FU chemotherapy, indicating that the chemotherapy tolerance and safety of old mice are remarkably reduced, which is consistent with clinical observation. In the current study, a 20% reduction in the dose of 5-FU was applied to the old mice as is often performed in clinical practices,^72^ which allowed us to see a clear protective effect by DR. As the toxicity of 5-FU is dose-related,^33^ we speculate using the same dose in the young mice might allow us to see a similar or stronger protective effect by DR. However, as the difference between DR and AL in young mice was already clear, an additional group of young mice using the same dose as the old mice was not set up in the current study. A limitation of the current study is that a murine tumor model was not used in conjunction to study the toxicity of 5-FU. A wealth of published studies have reported toxicities of 5-FU chemotherapy in murine tumor models, such as colorectal cancer and lung cancer models, with a total dose ranging from 120 mg/kg to 350 mg/kg per mouse.^37, 73–75^ The reported complications include: significant weight loss, diarrhea, intestinal mucositis, shortening and breakage of villus, loss of epithelial and crypt cells^37,73–75^ which were similar to the complications observed in the current study using a wild type mouse model with a dose of 5-FU at 200 mg/kg or 250 mg/kg per mouse. We speculate that a similar effect of DR would be observed in murine tumor models. Given that DR has been shown to prevent tumor development in various mouse models,^76–78^ it will be interesting to study the comprehensive effect of DR on chemotherapy in the context of murine mouse models in the future.

A growing body of studies showed that gut microbiota are intimately linked to the pharmacological effects of chemotherapies including 5-FU, and could modulate their therapeutic efficacy and toxicity.^16,17^ In the current study, we provide detailed experimental evidence that intestinal opportunistic pathogens, such as *P. mirabilis*, *E. coli, P. aeruginosa*, increase in number and translocate to distant organs in non-survived AL mice after chemotherapy, especially in old mice. Therefore, this study provides a clear model to show that the increase of intestinal opportunistic pathogens after chemotherapy and the translocation of these pathogens leading to severe infection could potentially be important reasons for the reduced safety and tolerance of chemotherapy and poor survival in elderly subjects.

This study also proposed for the first time that short-term DR before chemotherapy has a strong protective effect on the old mice and can significantly improve their survival after 5-FU chemotherapy. Previously, we have reported that DR can significantly reduce intestinal epithelial injury induced by high-dose methotrexate, which is related to the increase of intestinal *Lactobacillus*.^23^ However, the tolerance and safety of chemotherapy and the protective effect of DR on an old population receiving chemotherapy has not been studied. Moreover, the mucosal epithelial damage is a special side effect of methotrexate.^79,80^ Methotrexate is less commonly used in the treatment of solid tumors in clinical practice, since it is particularly metabolized renally and older patients frequently have renal insufficiency. In contrast, 5-FU is very widely used in malignant solid tumor chemotherapy.^81–83^ Although, there is a relative increase in the risk of use in patients with hepatorenal insufficiency, 5-FU is still widely used as a basic drug in the chemotherapy of old cancer patients due to its significant anti-tumor activity. Therefore, when compared to methotrexate, the 5-FU model is a more representative model for the study of mechanisms and exploring ways to increase safety and tolerance to chemotherapy of solid tumors in the elderly. Furthermore, in our previous study using the methotrexate model, clearing gut microbes with wide-spectrum antibiotics resulted in a worse survival and completely wiped out the rescuing effect of DR, whereas in the 5-FU model, administration of wide-spectrum antibiotics rescued survival of the *ad libitum* fed (AL) mice. These results indicated that 5-FU-induced death is mainly a result of a severe infection induced by intestinal opportunistic pathogens and involves all aspects of the intestinal barrier (physical, biological and chemical), while it is mainly the severe intestinal epithelium damage in the methotrexate model which resulted in the low survival of AL mice. Therefore, the current study provides new insights into understanding the mechanism of the toxicity of a fundamental chemotherapy drug for solid tumor treatment and a novel potential way to increase the safety and tolerance, especially for old individuals.

The current study provides a model reflecting the different types of intestinal barriers, physical, chemical and biological, in response to chemotherapy, which all have an important impact on chemotherapy tolerance, safety and survival. The current study provides the first evidence that the loss of lysozyme-positive cells (Paneth cells) in old AL mice after chemotherapy is an important mechanism that allows a dramatic increase of opportunistic pathogens in the intestine and their translocation which leads to infection of systemic organs and death. Notably, DR remarkably reduced intestinal inflammation, protected Paneth cells and maintained lysozyme, which increases *Lactobacillus* and inhibits amplification and translocation of intestinal opportunistic pathogens, so as to avoid infection and greatly improves the survival rate after chemotherapy.

Paneth cells are mainly regulated by inflammatory signaling pathways such as IFN-γ and TNF-α. Previous studies have shown that infection caused by gastric feeding of parasites can induce loss of Paneth cell loss.^84–86^ Inhibition of IFN-γ and TNF-α pathways can reduce the loss of Paneth cells which play an important role in resisting infection. It has been reported that Paneth cells were diminished after radiotherapy in response to inflammation.^87^ Our study found that DR significantly ameliorated the injury of intestinal epithelial cells after 5-FU chemotherapy and significantly inhibited the up-regulation of intestinal inflammatory factors such as IFN-γ and TNF-α. We speculated that the protection of physical barrier played an important role in protecting the loss of Paneth cells after chemotherapy.

Previous studies have shown that Paneth cells play an important anti-infective role in mouse models such as sepsis in which knocking-out Paneth cells significantly increased the mortality.^68,88,89^ Different bacteria taxa living in intestine have different sensitivity to lysozyme secreted by Paneth cells which can effectively kill intestinal opportunistic pathogens, such as *Proteus* and *E. coli*, whereas *Lactobacillus* is resistant to lysozyme.^67,68^ Previous studies have shown that increasing lysozyme could lead to a reduction in opportunistic pathogens in both *in vitro* and *in vivo* mouse models.^68,90,91^ Different bacterial taxa compete for nutrient acquisition in the intestine, with the inferior growth of certain bacteria can allowing for the dominant growth of other bacteria.^92,93^ Interestingly, our study found for the first time that lysozyme decreased after chemotherapy, especially in old mice. Our study shown that DR not only better preserved Paneth cells, but also regulated gut microbiota with significantly increased *Lactobacillus* and decreased *Proteus* and other opportunistic pathogens after chemotherapy, especially in old mice. We therefore speculate that this could be an important mechanism of how DR remarkably reduced the translocation of intestinal opportunistic pathogens and infection. Indeed, gastric gavage of lysozyme or *Lactobacillus* to AL mice reduced intestinal opportunistic pathogens and significantly improved the survival of mice treated with 5-FU, which further verified that lysozyme can increase Lactobacillus growth while significantly inhibiting the growth and translocation of intestinal opportunistic pathogens after chemotherapy. The results indicated that short-term DR before chemotherapy had a positive effect on the physical, chemical, and biological barriers of the intestine and improved survival of the mice after chemotherapy. It is still unknown how *Lactobacillus* is increased in DR mice and needs further investigation in the future.

Interestingly, we found that the decrease of lysozyme and the increase of opportunistic pathogens were more significant in elderly mice after chemotherapy. Previous studies have shown that the intestinal tissue of old mice exhibit minor histological changes in steady state.^8,10,94^ However, we and others have found that intestinal crypts display obvious defects of proliferation and differentiation in organoid culture and passaging in vitro.^11–13^ In the current study, we uncovered significant intestinal defects of old mice in response to chemotherapeutic stress in an in vivo mouse model, which could be significantly improved by short-term DR before chemotherapy. Moreover, the study also explored the impact of aging on gut microbiota in patients receiving 5-FU-based chemotherapy and found that opportunistic pathogens was increased in the feces of older patients (>65 years old) comparing to younger ones (<60 years old). Therefore, these results have significant potential in exploring ways to improve the safety and tolerance of chemotherapy in elderly patients.

In summary, the current study uncovered that 5-FU treatment led to a remarkable increase of intestinal opportunistic pathogens which could translocate to distant organs and cause infection and death in AL fed old mice. The loss of intestinal lysozyme seemed to play a key role in this process. DR for two weeks before chemotherapy can significantly protect against the loss of lysozyme and increase the content of Lactobacillus, resulting in a significant inhibition of intestinal opportunistic pathogens and their translocation, which lead to significantly improved survival of old mice receiving 5-FU treatment. Our study provides first experimental evidence that DR achieved a comprehensive protection of the intestinal mechanical, biological and chemical barriers, which significantly improved the overall survival of old mice. Our study reveals potentially important mechanisms for the poor chemotherapy tolerance of the elderly population, which can be significantly improved by short-term DR. The study presents DR as a novel way to potentially improve the patient prognosis, by improving the chemotherapy tolerance and safety of patients with malignant tumors.

## Supplementary Material

Supplemental Material
