# Supplementary material for: Dietary restriction rescues 5-fluorouracil-induced lethal intestinal toxicity in old mice by blocking translocation of opportunistic pathogens
Source: Gut Microbes. 2024 May 23;16(1):2355693. doi: 10.1080/19490976.2024.2355693 (PMC11123560; doi:10.1080/19490976.2024.2355693)
Supplement: Supplemental Material [file KGMI_A_2355693_SM9407.zip › Tang and Qiu et al_supplementary table 2,3.docx]

**Materials and methods**

**Quantitative Real-Time PCR (qPCR)**

Primer sets are listed in Table S2.

| Gene | Forward Primers | Reverse Primers |
| --- | --- | --- |
| β-actin | CTAAGGCCAACCGTGAAAAG | ACCAGAGGCATACAGGGACA |
| IL-6 | ACCAGAGGAAATTTTCAATAGGC | TGATGCACTTGCAGAAAACA |
| IL-1β | GATCCACACTCTCCAGCTGCA | CAACCAACAAGTGATATTCTCCATG |
| IFN-γ | GCCAAGTTTGAGGTCAACAAC | CCGAATCAGCAGCGACTC |
| TNF-α | CATCTTCTCAAAATTCGAGTGACAA | TGGGAGTAGACAAGGTACAACCC |
| IL-10 | GGTTGCCAAGCCTTATCGGA | ACCTGCTCCACTGCCTTGCT |

Primer sets are listed in Table S3.

| Gene | Forward Primers | Reverse Primers |
| --- | --- | --- |
| Lactobacillus | TGGAAACAGRTGCTAATACCGG | GTCCATTGTGGAAGATTCCC |
| Proteus | AAATTGTTGAATTAGCAGAAGCA | GCGATTGGGTGGATCAGTTC |
